# Production of Minor Ginsenoside CK from Major Ginsenosides by Biotransformation and Its Advances in Targeted Delivery to Tumor Tissues Using Nanoformulations

**DOI:** 10.3390/nano12193427

**Published:** 2022-09-30

**Authors:** Mohanapriya Murugesan, Ramya Mathiyalagan, Vinothini Boopathi, Byoung Man Kong, Sung-Keun Choi, Chang-Soon Lee, Deok Chun Yang, Se Chan Kang, Thavasyappan Thambi

**Affiliations:** 1 Graduate School of Biotechnology, College of Life Sciences, Kyung Hee University, Yongin-si 17104, Gyeonggi-do, Korea; 2 Department of Oriental Medicinal Biotechnology, College of Life Science, Kyung Hee University, Yongin-si 17104, Gyeonggi-do, Korea; 3 Daedong Korea Ginseng Co., Ltd., 86, Gunbuk-ro, Gunbuk-myeon, Geumsan-gun 32718, Chungcheongnam-do, Korea

**Keywords:** ginseng, ginsenoside, transformation, pharmacological activity, bioavailability, nanoformulation

## Abstract

For over 2000 years, ginseng (roots of *Panax ginseng* C.A. Meyer) has been used as a traditional herbal medicine. Ginsenosides are bioactive compounds present in ginseng responsible for the pharmacological effects and curing various acute diseases as well as chronic diseases including cardiovascular disease, cancer and diabetes. Structurally, ginsenosides consist of a hydrophobic aglycone moiety fused with one to four hydrophilic glycoside moieties. Based on the position of sugar units and their abundance, ginsenosides are classified into major and minor ginsenosides. Despite the great potential of ginsenosides, major ginsenosides are poorly absorbed in the blood circulation, resulting in poor bioavailability. Interestingly, owing to their small molecular weight, minor ginsenosides exhibit good permeability across cell membranes and bioavailability. However, extremely small quantities of minor ginsenosides extracted from ginseng plants cannot fulfill the requirement of scientific and clinical studies. Therefore, the production of minor ginsenosides in mass production is a topic of interest. In addition, their poor solubility and lack of targetability to tumor tissues limits their application in cancer therapy. In this review, various methods used for the transformation of major ginsenosides to minor ginsenoside compound K (CK) are summarized. For the production of CK, various transformation methods apply to major ginsenosides. The challenges present in these transformations and future research directions for producing bulk quantities of minor ginsenosides are discussed. Furthermore, attention is also paid to the utilization of nanoformulation technology to improve the bioavailability of minor ginsenoside CK.

## 1. Introduction

**Figure 1:**
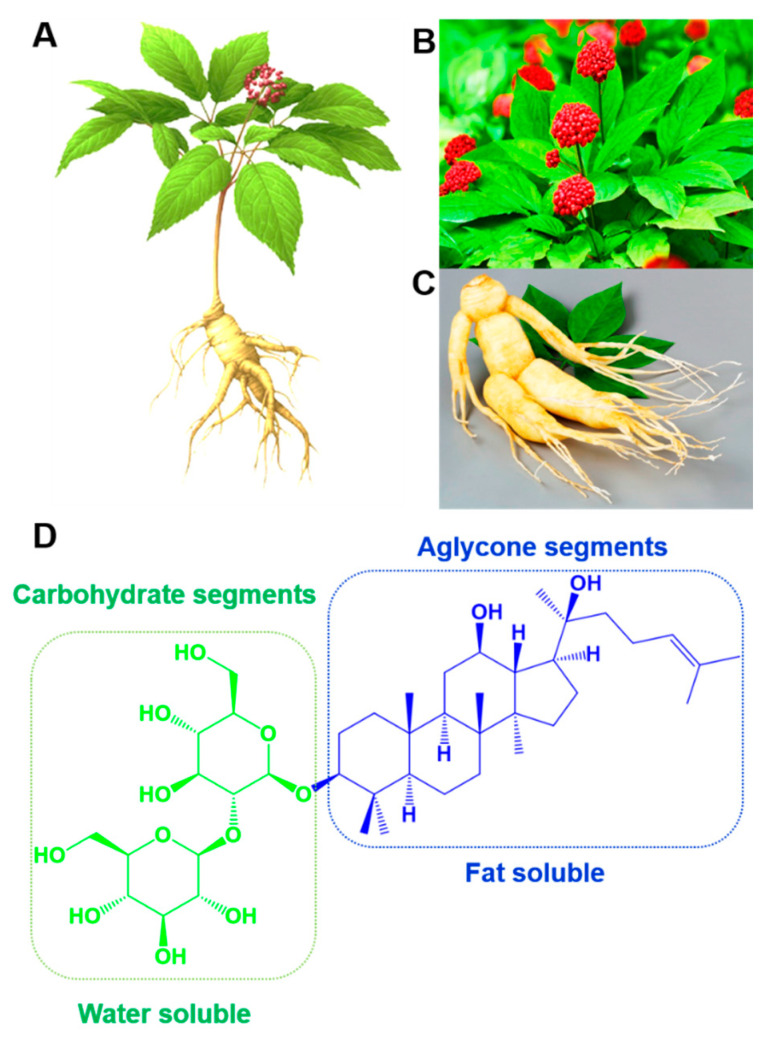
(**A**) Micrograph of *Panax ginseng* Meyer herb. (**B**,**C**) Berry and root of *Panax ginseng* Meyer herb. (**D**) Chemical structure of ginsenoside Rg3 consisting of water soluble carbohydrate and fat soluble aglycone segments.

**Figure 2:**
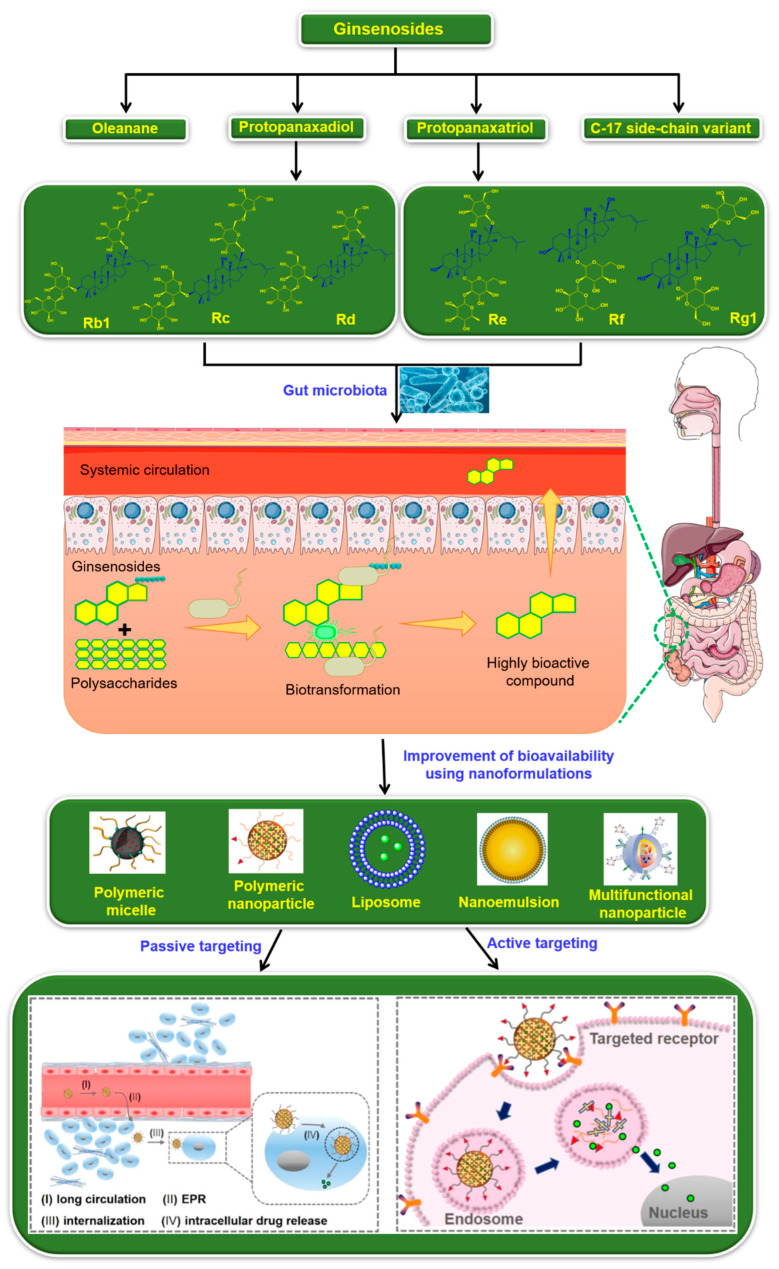
Schematic overview of classification of ginsenosides and their processing by gut microbiota. The gut microbiota effectively processes the ginsenosides into highly bioactive compounds which are then transfused through the systemic circulation. The bioavailability of ginsenosides is effectively improved by encapsulating into the nanoformulations and delivery of ginsenosides by passive or active targeting mechanisms.

The root of ginseng (*Panax ginseng* C.A. Meyer) has been used as a traditional herbal medicine for over 2000 years to treat various diseases, including cancer, diabetes and cardiovascular disease [1]. The presence of biologically active ingredients, such as saponins, polysaccharides, phenolic and nitrogenous compounds, in the ginseng roots are responsible for the pharmacological and biological activity [2]. Among them, ginseng saponins, called ginsenosides, are the major bioactive compounds and are responsible for the biological activity of ginseng (Figure 1) [3]. The major and most unique compounds of ginseng saponins (ginsenosides) are classified into dammarane-type, oleanane-type and ocotillol-type glycosides [4]. Based on the location and number of sugar units in the glycosides, ginsenosides are further divided into four types: protopanaxadiol (PPD), protopanaxatriol (PPT), oleanolic acid and C-17 side-chain variation type (C17SCV) (Figure 2) [5,6,7]. In general, PPD-type ginsenosides have sugar units at C-3 and/or C-20 positions [8]; whereas, sugar units are attached to C-3, C-6 and/or C-20 positions for PPT-type ginsenosides [9]. On the other hand, oleanolic acid-type ginsenosides [10] possess a glucuronic acid residue at the C-3 position, and C17SCV ginsenosides [11] possess variation in the C-17 position. Depending on their abundance, ginsenosides are classified as major and minor ginsenosides. Major ginsenosides account for more than 90% of the total ginsenosides; meanwhile, the remaining constitute minor ginsenosides and are present in miniscule amount [12]. Although both major and minor ginsenosides exhibit anti-oxidant, anti-diabetic, anti-inflammatory and neuroprotective properties, the extent of pharmacological effects are significantly high in minor ginsenosides when compared with major ginsenosides [13]. This can be explained by the fact that smaller molecular weights of minor ginsenosides effectively cross cell membranes, resulting in good absorption in the human body [14]. For instance, it has been reported that the anti-tumor activity gradually decreases as the number of sugar units increases in the glycosides [15]. Nevertheless, minor ginsenosides present in very low amount are insufficient to fulfill clinical and scientific research requirements. Therefore, it is important to prepare bulk quantities of minor ginsenosides through either simple transformation techniques from ginseng or from the transformation of major ginsenosides.

The major approach employed to produce minor ginsenosides is hydrolysis of major ginsenosides by physical, chemical and biological transformation [16]. Among them, physical and chemical transformations are considered as facile methods [17]. However, these approaches have limitations due to the formation of undesired by-products and lack of site-specificity. In addition, physical and chemical methods do not produce ginsenoside compound K (CK), as CK can be produced only through biotransformation [18]. Although biological transformations show higher specificity, enzymes used in these techniques are costly [19]. From this evidence, it is clear that each approach has its own advantages and disadvantages. In this report, we discuss physical, chemical and biological transformation methods used to prepare minor ginsenoside CK from major ginsenosides. It is known that the therapeutic potential of CK is often limited due to its poor aqueous solubility, membrane permeability and bioavailability [20]. To surmount these issues and improve the therapeutic potential of CK in cancer therapy, various nano-based delivery systems have been used to load and specifically deliver CK into tumor tissues [21].

A variety of nanoformulations, including polymeric nanoparticles, nanoemulsion and liposomes, have been employed as delivery systems for CK [21]. These nanoformulations can reach tumor sites via either passive (enhanced permeability and retention (EPR)) or active (tumor-specific ligand and receptor interaction) targeting mechanisms [22,23]. After internalization, the nanovehicles can release CK and increase the anti-tumor effect. In this review, we first discuss the biotransformation of CK using different methods, then, we highlight the nano-based delivery approaches used to deliver CK and highlight the progress made in tumor treatment, which could be useful in designing optimal delivery systems for CK.

## 2. Transformation

**Figure 3:**
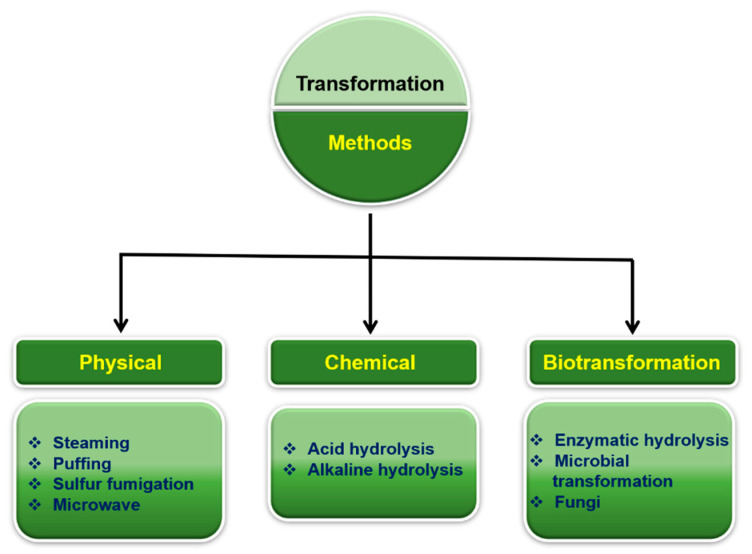
Transformation of ginsenosides using physical, chemical and biotransformation methods. These methods transform a ginsenoside into another ginsenoside by altering the chemical structure of the ginsenoside.

Owing to the remarkable pharmacological properties of minor ginsenosides, research has focused on the transformation of major ginsenosides into minor ginsenosides by hydrolysis (Figure 3). Physical and chemical transformation methods, such as thermal and hydrolysis (acid or basic) methods, have often been employed. In addition, biotransformation methods such as enzymatic and microbial conversion modalities have also been employed for the transformation. In this section, we discuss the major methods used for the conversion of major ginsenosides into minor ginsenosides.

### 2.1. Physical Methods

#### 2.1.1. Steaming

Primordially, steaming is the traditional way of processing ginseng products [24]. The steaming of ginseng was performed for 3 h at 90–100 °C, and to remove the moisture, drying was carried out by dry oven [25]. Because of this processing condition, the pharmacological activities of ginseng may vary. Hence, if the steaming temperature increases, the composition of ginsenosides is affected. During this steaming process, some of the major ginsenosides were converted into minor ginsenosides. Likewise, fresh ginseng has malonyl ginsenosides which were de-malonylated and de-glycosylated during steaming, and further transformed into other ginsenosides, such that ginsenosides Re, Rg1 and Rd were transformed into ginsenosides Rg2, Rh1 and Rg3 [26]. Hence, this shows that the heating process of ginseng produces malonyl ginsenosides which are not found from the steaming process. The ginsenosides Rg3, F4 and Rg5 were produced only if the steaming process of ginseng was performed at 120 °C [27], but the naturally occurring ginsenoside content decreased. In conjunction with this, fresh ginseng roots were steamed using an autoclave which resulted in the increase of the ginsenoside content at 98 °C, whereas it decreased for 120 °C steaming. Moreover, the content of ginsenosides changed with respect to the incubation time. Therefore, it is important to control time and temperature during the steaming process. Furthermore, the naturally occurring ginsenoside content deteriorated during the thermal process.

In general, controlled heat treatment of ginseng produces a larger amount of rare ginsenosides than conventional methods. Often, red ginseng was produced by steaming white ginseng for 3 h at 98 °C or drying for 12 h at 60 °C. It is well known that chemical structures and biological activities of ginsenosides are changed when they are steamed at high temperature (~100 °C). Therefore, steaming temperature and time are more important to control to avoid the structural damage of ginseng products. Studies on controlling the steaming process to prepare minor ginsenosides have been reported. For instance, steaming of ginsenosides at 120 °C produced rare ginsenosides such as F4, Rg3 and Rg5; these ginsenosides are not present in raw ginseng [28]. Unfortunately, during the steaming process, the contents of other major ginsenosides Rb1, Rb2, Rc, Rd, Re and Rf were decreased. The content of total ginsenosides was increased when the ginseng roots were steamed at 98 °C. During these processes, steaming and heating time also play an important role in controlling the content of ginsenoside products.

#### 2.1.2. Puffing

Puffing is a process of expanding or breaking/bursting a preexisting structure by liberating the gas within the structure [29]. There are two different types of puffing which are commercially used, namely gun puffing and oven puffing [30]. The gun puffing process has been widely used, and produces a gradual increase in the apparent volume of six to eightfold ginsenosides, whereas the oven puffing process is not as efficient as the gun puffing process and uses a high-temperature short-time (HTST) treatment with heated sand to produce ginsenosides under three to six-fold. On the whole, due to dehydration, a given sample is expanded and this leads to the quick diffusion of water vapor out of it. Raw ginseng was steamed and dried under specified conditions to produce white, red and black ginseng, which underwent the puffing process with rice to avoid burning. Once the puffing process was completed, the puffed ginseng samples and the rice were separated and cooled for some time and stored in a refrigerator. The thermal and non-thermal extraction of the puffed and non-puffed ginseng were prepared. After the puffing process of ginseng, the naturally occurring ginsenosides were comparatively decreased due to the increased puffing pressure, and the ginsenoside content was higher than the non-puffed ginseng (control). Importantly, there was some modification in the chemical structure of the ginsenosides, which in turn, signifies that even though the puffing process of ginseng gave higher yields of extraction, it could influence the ginsenoside composition.

#### 2.1.3. Sulfur Fumigation

A desiccator is an airtight container for maintaining an atmospheric low humidity using an appropriate drying agent, which is usually placed in the bottom part of the container. Here, sulfur is used as the drying agent and ginseng is place at the top of the desiccator. The sulfur is fired and the desiccator is closed, whereas the container is filled with gas. The gas is emitted after some time. This process continues until the ginseng is completely dried out. This process of involving sulfur as the drying agent via burning is called sulfur fumigation [31,32]. This way of processing ginseng has its own benefits and disadvantages. More importantly, this way of processing ginseng pollutes the atmosphere and also reduces the quality of ginseng. Even though this method preserves the ginseng as fresh as ever, it may also have the chance of presenting the decomposed ginseng as fresh ginseng after the fumigation process. It also produces SO_2_ through the amalgamation of sulfur and oxygen, which in turn, produces sulfurous acid through the amalgamation of SO_2_ and water molecules. This sulfurous acid presents the color of the ginseng as that of fresh ginseng. This process also has disadvantages such as producing damage in bioactive compounds which degrade the ginseng’s quality [33]. The most important issue in this method is the production of harmful substances, such as high levels of SO_2_, on the ginseng.

#### 2.1.4. Microwave Conversion

The conventional methods for the conversion of ginsenosides have some disadvantages such as degradation of ginsenosides and a longtime heating requirement. To overcome these drawbacks and to improve the preservation of the herb and its bioactive constituents, the methods of heating and drying herbs is important. Hence, microwave oven heating of ginseng gives efficient results, which are simple to process and are time saving [34]. The ginseng extract solution was kept in a microwave and the ginsenoside degradation process was performed at 165 °C for 5 min. After the degradation process, water saturated n-butanol was used for extraction and then evaporated. Finally, the ginsenosides Rh2 and Rg3 were the outcome of the major ginsenosides [35]. It was reported that PPD- and PPT-type ginsenosides were efficiently increased during the microwave heating process [36]. Moreover, the quality of ginseng was not affected during microwave heating. Recently, it was also reported that the trace amount of ginsenosides Rk1 and Rg5 increased by the microwave processing method [37]. These ginsenosides have natural sugar steroids which inhibit the metastasis of lung cancer.

### 2.2. Chemical Methods

#### 2.2.1. Acid Hydrolysis

It is well known that under acidic conditions, major ginsenosides are decomposed into rare ginsenosides [38]. For instance, HCl has been used to treat the ginsenosides for 120 min at 37 °C. The resultant mixtures were tested with chromatography in which the prosapogenins were not found since ginsenosides decomposition occurred. It has been stated that the transformation of ginsenosides is possible only when it is treated under acidic conditions, and not in neutral conditions. Among different concentrations of formic acid used to treat the ginsenosides for conversion, 0.01% of formic acid gave an increased ginsenoside yield [39]. Specially, ginsenoside mixtures treated with formic acid gave numerous ginsenosides. Other than formic acid, there are some other acids such as tartaric acid, lactic acid and citric acid which are used for the chemical transformation of the ginsenosides. In particular, PPD ginsenosides are better in chemical transformations under mild acid conditions. The production of specific ginsenosides such as Rg3 increased with the effective temperature at 60 °C and time of 5 h. HCl provided the highest transformation of ginsenosides compared to lactic acid and citric acid. Hence, it has been reported that the transformation of ginsenosides was easily completed under acidic conditions, yet it was noted to have control over the pH, temperature and time of the transformation.

#### 2.2.2. Alkaline Hydrolysis

Acid hydrolysis is a chemical method used to decompose ginsenosides. Just as acid hydrolysis has its own way of degrading ginsenosides, so too does the alkaline hydrolysis of ginsenosides. The transformation of ginsenosides by alkaline hydrolysis is mainly used for the structural elucidation of saponins and to analyze the content of the saponin group as well as sugar residues [40]. This ginsenoside transformation technique happens under the condition of high pressure, high temperature and high pH in order to obtain minor ginsenosides. It was reported that the hydrolyzing of ginsenosides was performed using a boiling water bath for 8 h with NaOH (2 M), and the newly obtained product ginsenoside Rh19 was extracted using ethyl acetate [41]. Since PPD-type ginsenosides were processed with alkaline hydrolysis using isoamyl alcohol as the solvent for 24 h, as the time increased to 32 h with the same conditions, the yield of PPD-type ginsenosides was degraded [42]. It has been found that the yield of PPD-type ginsenosides is degraded when prolonged heating is performed.

#### 2.2.3. Deep Eutectic Solvent (DES) with Choline Chloride (ChCl)

DES has proved a substitute for the traditional organic solvent used in biocatalytic processes [43]. It has numerous benefits such as less vapor pressure, high thermal stability, easy recyclability, electrical conductivity and non-flammability, and more importantly it contains good solubility for various compounds due to the presence of a hydrogen bond donor and hydrogen bond acceptor. Because of these properties, DES is used for enzymatic conversion medium. Its viscosity is to be noted as a drawback as the enzymatic reaction alters during the conversion process even though it is a liquid in room temperature. It has been proven that ChCl as a buffer solution reduces the viscosity of DES by ten times, even after the temperature has been raised up to 50 °C, and in turn, the conversion of natural products takes place without creating any enzymatic denaturation. Moreover, it was noted that DES was 1.5 times better than PBS treatment. From the rhizosphere soil of ginseng, *Talaromyces purpureogenus* was obtained, which catalyzed the manufacturing of β-glucosidase with high purity [44]. On the research on finding the best DES, a variety of DESs were tested based on their stability and activity of β-glucosidase, and a finalized DES was found as a reaction medium in the ratio with ChCl and ethylene glycol (EG) of ChCl:EG = 2:1 to transform ginsenoside Rb1 into CK [45]. With this proportion, the β-glucosidase half-life was increased to 96%. The conversion rate of CK was 80.6% after transformation at 60 °C for 48 h with pH 4.5 [46]. The transformation pathway of CK was explored once the transformation was verified with the kinetics of the enzyme and structure conformation, and the conditions for the enzymatic reaction such as reaction time, temperature, pH and substrate concentration were optimized. The same way of enzymatic hydrolysis of ginsenoside Rb1 has been tried with Aspergillus terreus. The conversion rate of Rb1 to CK was improved to 91.3% from 80.6% found last time [47]. Later, this reactive medium, with the combination of DES and ChCl, was termed ATPS (aqueous two-phase system) to verify the components of the DES associated with the hydrogen bond based on the ChCl. This also confirmed that this process provides non-toxic and immunological responses. Once the process of separation was completed, the transformed ginsenoside CK was obtained from both the top phase (DES) and bottom phase (salt solution), and its obtained percentage was 75.79% at 55 °C with pH 5.0; at the same time 61.14% of β-glucosidase was also recovered.

### 2.3. Biotransformation

Most of the minor ginsenosides, such as Rh1, Rh2, Rg3, F2 and compound K, are prepared from major ginsenosides [48]. In particular, CK is a non-natural ginsenoside generated from PPD-type ginsenosides by biotransformation [49]. Owing to the good pharmaceutical properties of CK, attention has been paid to the conversion of major ginsenosides into minor ginsenosides via physical and chemical transformation techniques [50]. However, these transformation methods induce side reactions. In particular, chemical transformations of ginsenosides lead to unwanted side reactions such as epimerization and hydroxylation [51]. In addition, these techniques are not green methods and create environmental pollution. On the other hand, biotransformation of ginsenosides using enzymatic and microbial methods shows good selectivity, mild reaction conditions and is environmental-friendly [52].

#### 2.3.1. Enzymatic Transformation

Enzymatic transformation of ginsenosides is highly regiospecific and has been found to be a promising method to obtain minor ginsenosides [53]. For instance, CK is produced from ginseng root extract via enzymatic transformation using various enzymes such as pectinase, lactase and β-glycosidase [54]. Since the ginsenosides are comprised with a dammarane-type skeleton, some special β-glucosidases can specifically hydrolyze the ginsenoside-β-glucoside linkages [55]. Thus far, numerous β-glucosidases enzymes have been reported with the specific capability to transform ginsenosides into CKs [56].

The pioneering work performed by Park et al. reported the purification and characterization of β-glucosidase *Fusodobacterium* K-60 from human intestinal feces [57]. The *Fusodobacterium* K-60 enzyme specifically hydrolyzes the ginsenoside Rb1 into CK. However, the biotransformation of Rb1 to CK is weak and is not optimal for scaled up process. To improve the conversion rate of Rb1 into CK, Qin et al. purified a novel ginsenoside-hydrolyzing β-glucosidase from *Paecilomyces Bainier* sp. 229 using chromatography [58]. The maximal enzyme activity was observed when the ginsenosides Rb1 were incubated with the enzyme at 45 °C and pH 3.5. One day after the incubation, approximately 84.3% of the ginsenoside Rb1 was converted to CK by the following pathway: Rb1 → Rd → F2 → CK. In addition to these enzymes, recombinant β-glucosidase enzymes such as *Terrabacter ginsenosidimutans* sp. [59], *Escherichia coli* [60] and *Aspergillus Niger* have also been reported to transform Rb1 into CK.

**Figure 4:**
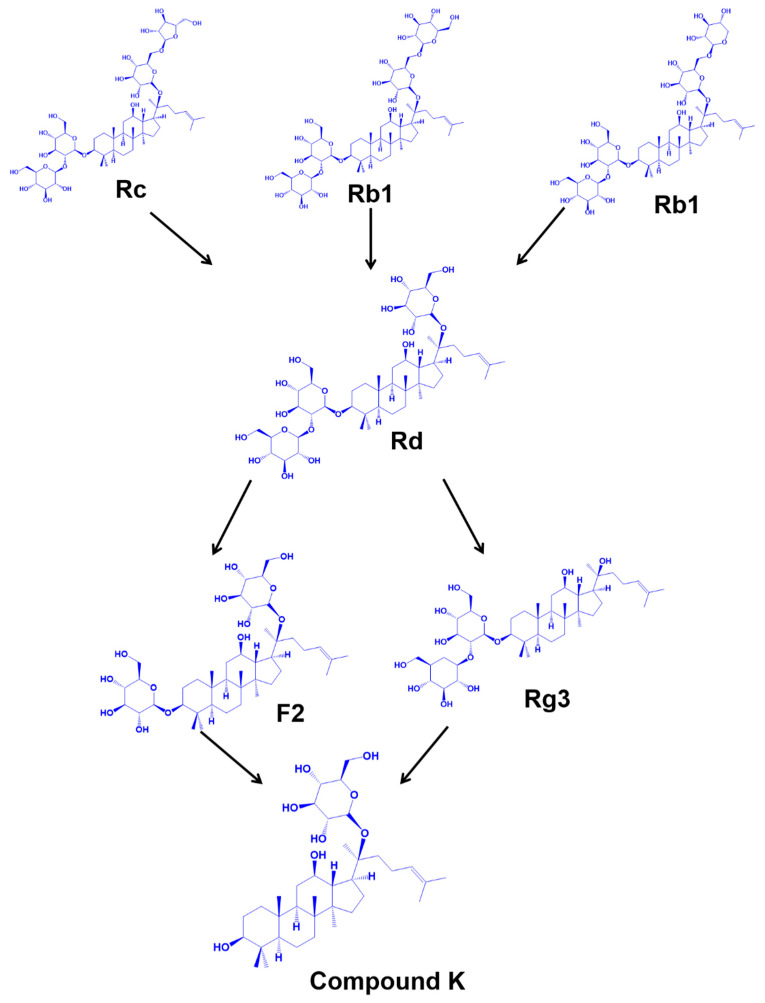
Biotransformation of major ginsenosides into more active CK.

β-glycosidases are alternative to β-glucosidases, and have often been used for the hydrolysis of ginsenosides [61]. β-glycosidases specifically hydrolyze the PPD-type ginsenosides [62]. Noh et al. reported the preparation of CK from ginseng root extract using β-glycosidases produced from *Sulfolobus solfataricus* [63]. They reported the transformation of Rb1, Rb2, Rc or Rd to CK by two transformation pathways: (1) Rb1 or Rb2 → Rd → F2 → compound K, and (2) Rc → compound Mc → compound K (Figure 4). Although this method showed good specificity, the conversion rate of ginsenoside to CK was poor. Therefore, recombinant β-glycosidase from *Microbacterium esteraromaticum* [64] and *Pyrococcus furiosus* [65] has been developed for the transformation of major ginsenosides into minor ginsenosides. The recombinant *Pyrococcus furiosus* had high productivity in converting Rd to CK with a yield of 83% [65].

In addition to the pure enzymes, crude enzymes were screened to examine their hydrolytic activities against ginseng extract [66]. A total of 46 crude mixtures from different organisms were reported in which α-amylases, β-amylases, emulsin and hesperidinases showed weak hydrolyzing properties [67,68,69]; whereas, cellulases, naringinases and pectinases exhibited reasonable hydrolyzing properties [68]. Cellulase from *Trichoderma viride* was shown to have high hydrolytic activities to yield CK from PPD ginsenosides in large quantities. The biotransformation method used to prepare CK offers advantages as this method is facile and no organic solvents are required.

#### 2.3.2. Microbial Transformation

In general, microbial transformation is considered to be a major method to prepare CK [70]. It includes the use of crude enzymes from *Lactobacillus paralimentarius* [71], *Fusarium sacchari* [72], *Acremonium strictum* and *Caulobacter leidyia* [67]. For instance, the metabolic transformation of CK from ginsenosides Rb1, Rb2 and Rc by intestinal flora was systematically investigated by Hasegawa et al. [73]. Upon anaerobic incubation of ginsenosides Rb1, Rb2 and Rc with human intestinal microflora, the ginsenosides were converted into CK. Among them, bacterial strains, such as *Bacteroides* sp., *Bifidobacterium* sp. and *Eubacterium* sp., isolated from human intestinal feces, effectively transformed Rc into CK [74]. Intestinal bacterial metabolism of ginsenoside Rb1 degradation into CK was mainly dependent on the gut microbiota composition.

Similar to the bacteria, fungus is easy to culture and can replace human intestinal bacteria for obtaining CK via biotransformation [72]. Zho et al. prepared CK cost effectively from *Panax notoginseng* saponins (PNS) using fungal biotransformation [75]. The same team also reported *Paecilomyces bainier* sp. 229 fungus to effectively convert PNS into CK [76]; the conversion rate of CNS to CK was significantly higher than the previous rate (82.6% vs. 35.4%). In addition, fungus generated from soil-cultivated ginseng, including *F. sacchari* [72], *A. strictum* [77], *A. niger* [78] and Fusarium moniliforme [79], showed good biotransformation of major ginsenosides into minor bioactive ginsenosides. However, these fungi are not approved as standard food-grade microorganisms. Therefore, researchers focused on the development of food-grade edible microorganisms, *Aspergillus usamii, Bifidobacterium* sp. and *A. niger*, that could transform ginsenoside Rb1 into CK via Rd → F2 → compound K [78]. Various strains extracted from Kimchi, a Korean staple food, such as various *Leuconostoc* strains, showed good transformation of PPD-type ginsenosides into CK [47].

The biotransformation of major ginsenosides into minor bioactive compounds has often been performed using microorganisms. In addition, a synthetic biology strategy has also been employed for transformation. The metabolically engineered yeast encoding the heterologous UGTPg1 gene could readily transform PPD ginsenosides into CK [80]. Yeast strain harboring CYP716A47 and UGTPg1 genes have shown good biotransformation of CK. A summary of different transformation methods that enlist transformation pathways, advantages and disadvantages of ginsenoside transformations is given in Table 1.

## 3. Improved Pharmacological Effects Using Nanoformulations

Despite the outstanding health and pharmacological benefits, the applications of ginsenosides have been severely limited because of their poor bioavailability [81]. It has been reported that the bioavailability of orally administered ginsenosides was found to be below 5% [21]. The poor availability of ginsenosides is attributed to the poor permeability, lack of membrane permeability and degradation of ginsenosides in gastrointestinal tract fluids [81,82]. Therefore, the bioavailability of ginsenosides can be improved by creating them in a soluble form, either by coating with polymers or increasing the glucose units, that could protect ginsenosides effectively from the physiological environment and direct the ginsenosides to target sites of interest [82]. Numerous nanoformulations, such as nanoemulsions [83], polymeric particles [84,85], liposomes [86], exosomes [87], mesoporous silica nanoparticles [88] and other miscellaneous formulations have been developed to improve the pharmacological efficacies of ginsenosides [89,90,91]. These formulations can be used to load ginsenosides and administered to the body by various administration techniques to improve the bioavailability of ginsenosides. The nanoformulations effectively solubilize ginsenosides and improve the dissolution rate and absorption during blood circulation [92]. More importantly, nanoformulations facilitate the permeation of ginsenosides to the cellular membranes.

To prepare the nanoformulations, the fundamental materials used to prepare the formulations should be non-toxic, biocompatible and biodegradable [93,94,95,96,97,98,99,100]. In addition to these characteristics, nanoformulations should not have any undesirable interactions with ginsenosides, which can minimize the reduction in the bioavailability of ginsenosides. More importantly, after performing their role as a delivery carrier, the nanoformulations should be eliminated from the body or biodegraded into non-toxic fragments [101,102,103].

Often, to control the release of ginsenosides from delivery systems, the selection of delivery formulations plays an important role [21]. Based on the ginsenosides and their physicochemical characteristics, the structure and morphology of nanoformulations should be carefully chosen to improve the bioavailability of ginsenosides and minimize toxicities [104].

### 3.1. Nanoemulsions

Nanoemulsions are clear, stable and heterogenous dispersions of two immiscible liquids (e.g., oil-in-water or water-in-oil) with a droplet size ranging from 20 nm to 600 nm [105]. Often, nanoemulsions are stabilized by amphiphilic surfactants and exhibit enhanced long-term physical stability. Unlike other nanocarriers, nanoemulsions are not influenced by destabilization phenomena such as sedimentation and coalescence [106]. Nanoemulsions are prepared using high-pressure homogenizers and ultrasound generators by applying high mechanical energy. In recent years, nanoemulsions paid great attention to topical drug delivery in treating various diseases such as local infections, psoriasis and skin cancer [107]. The hydrophilic outer shell of nanoemulsions has good contact with the skin surface, and the presence of surfactants can enhance the skin tissue penetration. In addition, the small size of nanoemulsions effectively increases the drug absorption rate and can increase the bioavailability of drugs. Ginsenosides are known to cure various skin-related diseases, therefore, loading them into nanoemulsions could improve their bioavailability [108]. Xue et al. utilized a water-in-oil emulsion technique to load CK into the aldehyde-modified β-cyclodextrin (β-CD-CHO) [109]. The controlled emulsion technique showed over 70% encapsulation efficiency, which implied good loading capacity of the emulsion technique. The CK-loaded β-CD-CHO reacted with amine-modified carboxymethyl cellulose and formed nanogel via the Schiff base reaction. The CK-loaded nanoemulsions effectively killed the A549 cells when compared with free CK.

### 3.2. Polymeric Nanoparticles

Polymeric nanoparticles are generally referred to as small colloidal solids with a particle size range from 10 to 1000 nm [110,111,112,113,114]. Owing to their tunable size, polymeric nanoparticles can solubilize large quantity of drugs and improve their absorption. Such physically entrapped drugs can control the release of drugs; more importantly, polymeric nanoparticles effectively protect the drugs from physiological environments. It has also been known that small-sized particles selectively accumulate into the tumor site via the EPR retention effect [97]. A novel PPD-type ginsenoside, 25-OCH_3_-PPD (GS25), has been known to exhibit remarkable anti-cancer activity in vitro. However, the application of GS25 has been limited owing to its hydrophobic nature. To utilize GS25 without minimizing its anti-cancer activity, Voruganti et al. utilized traditional polyethylene glycol (PEG)-poly (lactic-co-glycolic acid) (PLGA) nanoparticles to load GS25 and improve its oral bioavailability [115]. The inhibitory effects of GS25-loaded nanoparticles on mouse double minute 2 (MDM2), a negative regulator of the p53 tumor suppressor, were examined in various preclinical models. GS25-loaded polymeric nanoparticles exhibited better MDM2 inhibition and improved oral bioavailability and enhanced in vitro and in vivo activities.

**Figure 5:**
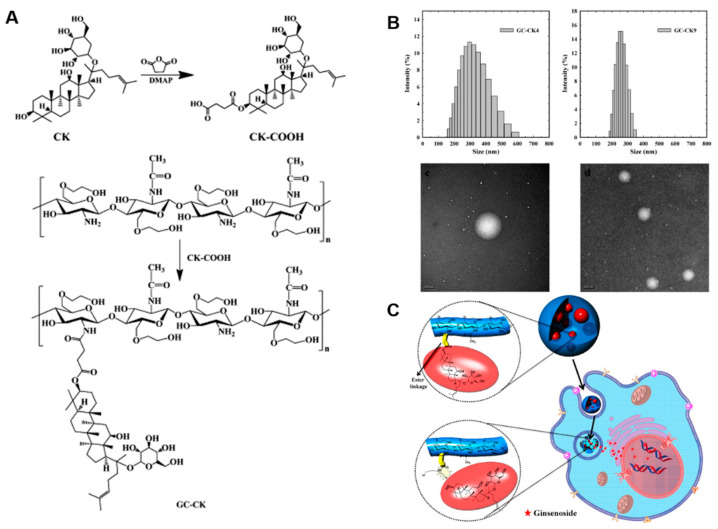
(**A**) Synthesis of compound K-bearing glycol chitosan conjugate. (**B**) Particle size distribution and morphology of compound K-bearing glycol chitosan conjugate with different degrees of substitution of CK to the backbone of glycol chitosan. (**C**) Schematic illustration of pH-responsive release of CK from the conjugate. Reprinted with permission from Ref. [116]. 2014, Elsevier Ltd.

In general, solubility, stability and non-specific toxicity of bioactive agents including ginsenosides can be improved by conjugating hydrophilic polymers. For instance, Mathiyalagan et al. reported the solubility improvement of CK by conjugating it with glycol chitosan (GC) (Figure 5) [116]. The CK was conjugated to the backbone of GC through an amide bond formation. The GC–CK conjugates formed self-assembled nanoparticles and exhibited stability in the physiological condition. Interestingly, the solubility of CK was increased over 10-fold after conjugation with GC. The GC–CK conjugates were effectively dissolved in mild acidic environments due to the presence of the ester linkage between GC and CK. In vitro toxicity studies showed that GC–CK conjugates enhanced anti-cancer activities in various cancer cell lines when compared with CK alone. This study suggests that polymer conjugation to CK could be a viable option to improve solubility and bioavailability.

**Figure 6:**
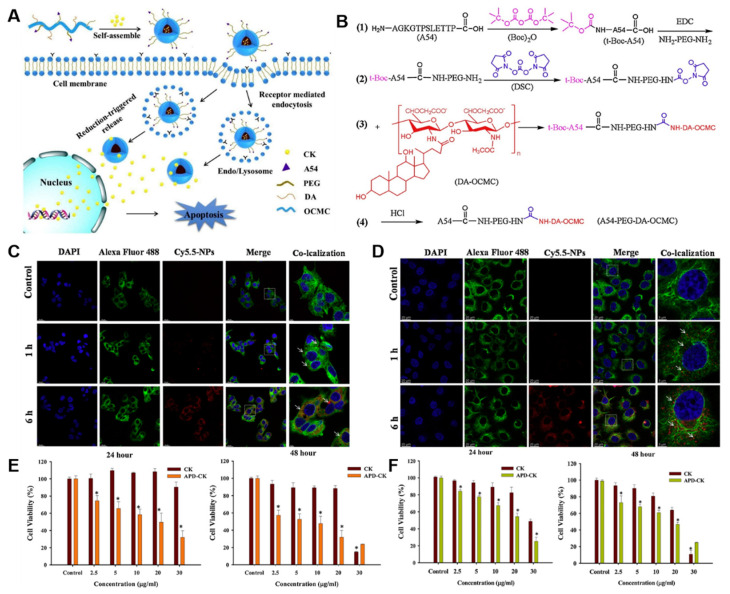
(**A**) Structural pathway of APD-CK inside the cell. (**B**) Conjugation of polymer (A54-PEG-DA-OCMC) before loading ginsenoside CK. Cellular uptake of APD-CK nanoparticles after incubating for 1 h and 6 h with (**C**) HepG2 and (**D**) Huh-7 cells. (**E**) In vitro cytotoxicity of normal ginsenoside CK and APD-CK against HepG2 cell line. (**F**) In vitro cytotoxicity of normal ginsenoside CK and APD-CK against Huh-7 cell line. Asterisks (*) denotes statistical significance difference compared with control group. Reprinted with permission from Ref. [117]. 2020, Elsevier Ltd.

Zhang et al. prepared tumor-homing A54-peptide-conjugated nanoparticles for targeting the liver (Figure 6) [117]. The nanoparticles were prepared using the self-assembled conjugate of deoxycholic acid-O-carboxymethyl chitosan, and the average size of the nanoparticles was 171 nm. The nanoparticles effectively loaded and controlled the release pattern of CK. From in vitro cytotoxicity studies, the CK-loaded nanoparticles (APD-CK) exhibited enhanced cytotoxicity against HepG2 and Huh-7 cells. The effective targeting characteristics of chitosan-based nanoparticles could effectively target and treat liver cancer. In vitro release studies demonstrated a pH-responsive release pattern of CK. HepG2 and Huh-7 cells exposed with APD-CK nanoparticles showed an enhanced anti-tumor effect compared to free CK. These results suggest the potential of site-specific delivery of CK using nanoformulations, which subsequently improves the anti-tumor activity of CK.

Conjugation of ginsenosides with inert polymers requires a large amount of nanocarriers to be administered into the body in order to obtain an anti-tumor effect. The large number of inert polymers administered to the patients is toxic. Therefore, development of nano-sized particles using only bioactive agents without inert polymers may reduce the toxic and harmful ingredients and also reduce the dose prescriptions during treatments [118]. Dai et al. prepared ginsenoside nanoparticles by combining them with chemotherapeutic agents such as hydroxycamptothecine, dihydroartemisinin and betulinic acid [119]. The resulting nanoparticles exhibited enhanced anti-tumor activity due to the synergism. In particular, the nanoparticles exhibited better tumor selectivity, longer circulation times and an anti-tumor effect in the tumor xenograft model. From these results, it is clear that encapsulation of ginsenosides or conjugation of ginsenosides using polymers can improve their anti-cancer effect.

### 3.3. Liposomes

Liposomes are lipid bilayer-based vesicular delivery systems which have been extensively studied because of their high stability, long-term circulation in the blood stream and structural mimicking characteristics with biological membranes [120]. Liposomes have an aqueous core that is separated by a hydrophobic membrane [121,122,123]. Owing to this unique feature, liposomes can encapsulate both hydrophobic and hydrophilic drugs. The large surface area and nanometer size of liposomes increase the absorption rate of the encapsulated drugs. Furthermore, liposomes not only protect encapsulated drugs from burst release during systemic circulation, but also protect the drug from the harsh acidic environment of the stomach during oral delivery. Since liposomes are prepared using phospholipids, they are biocompatible and biodegradable and can be used for systemic administration. Therefore, liposomes have been utilized to alter the bioavailability of various therapeutic agents including ginsenosides.

**Figure 7:**
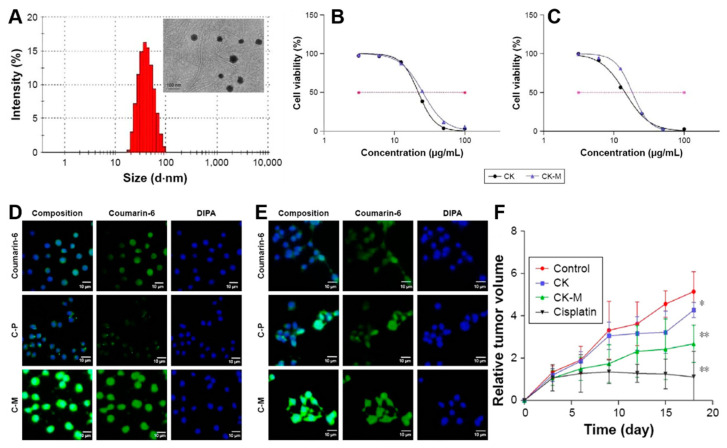
(**A**) Size distribution of the formulated CK-M and the spherical morphology of the CK-M micelle in TEM. (**B**,**C**) In vitro cytotoxicity of free ginsenoside CK and CK-M against A549 and PC-9 cell lines. (**D**,**E**) Cellular uptake of C-P (coumarin-6-loaded PEG-PCL micelles), C-M (coumarin-6-loaded mixed micelles) and coumarin-6 dyes under the fluorescent microscope in A549 cell line and in PC-9 cell line. (**F**) Relative tumor volume was calculated in vivo with cisplatin as positive control (* *p* < 0.05, ** *p* < 0.01). Reprinted with permission from Ref. [125]. 2017, Dove Medical Press.

Yang et al. prepared CK-loaded liposomes to improve the solubility and tumor targeting characteristics of CK [124]. The CK-loaded liposomes were prepared by combining D-α-tocopheryl polyethylene glycol 1000 succinate (vitamin E or TPGS), phospholipid and CK using the thin film rehydration technique. The liposomal formulation showed over 98% encapsulation efficiency of CK. The CK-loaded liposomes exhibited enhanced cellular uptake in A549 lung cancer cells. As a result, the anti-tumor activity of CK-loaded liposomes was significantly higher when compared with free CK in the A549 xenograft tumor. In a similar approach, the stability of the nanoparticles was further improved by introducing amphiphilic co-polymers (PEG-PCL), which showed better inhibition of A549 tumors (Figure 7) [125].

**Figure 8:**
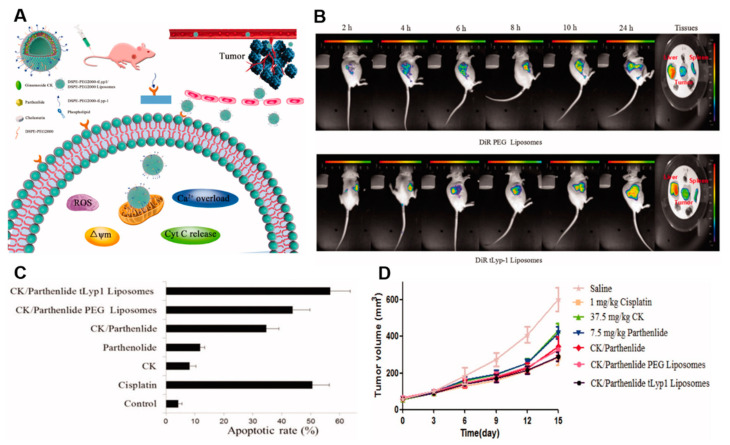
(**A**) Anti-tumor pathway of CK/tLyp-1 liposome. (**B**) In vivo process of CK/tLyp-1 liposome and its active targeting. (**C**) A549 treated with CK/tLyp-1 liposome shows higher apoptotic rate. (**D**) In vivo measurement of tumor volume. Reprinted with permission from Ref. [126]. 2018, Taylor & Francis Online.

The anti-tumor activity of CK can also be improved by specific targeting of liposomes by introducing targeting ligands. For instance, Jin et al. developed a novel tumor-homing peptide, tLyp-1, containing liposomes for the tumor-targeted delivery of CK (Figure 8) [126]. In addition to the CK, parthenolide, an anti-cancer agent known to induce anti-cancer activity by inhibiting the phosphoinositide 3-kinase/protein kinase B signaling, was also loaded to enhance anti-tumor activity [126]. The combination formulation of parthenolide and CK containing liposomes exhibited synergistic anti-cancer effects. The liposomal formulation prepared with CK and parthenolide showed an enhanced anti-tumor effect compared to the liposome formulation prepared with a single drug in the subcutaneous A549 tumor model.

These results imply that distinctive characteristics of liposomes effectively protect the ginsenoside CK during circulation and improve the bioavailability. Some of the liposome formulations, such as liposomal doxorubicin, vincristine liposome, irinotecan liposome and paclitaxel liposome have been used in clinical trials, which indicates the future prospects of CK-loaded liposomes in cancer therapy [127].

### 3.4. Miscellaneous Formulations

Although conventional liposomes have shown good pharmacological effects, their applications are limited to systemic administration. Often, ginsenosides are used for dermal applications; therefore, nanoformulations that penetrate the skin can enhance the topical applications of ginsenosides [128]. In recent years, to improve skin penetration in the topical delivery of ginsenosides, advanced forms of liposomes such as ethosomes, transfersomes and niosomes have been developed [129]. Each of these formulations contains some specific characteristics or portions that give skin permeability to the formulations. For instance, the presence of ethanol in the ethosomes provides membrane flexibility and is involved in disrupting the skin barrier [130]. On the other hand, transfersomes consist of edge activators or surfactants that effectively involve penetrating the skin tissues [131]. Few studies have shown encapsulation of ginsenosides, and their application in topical delivery has been examined. However, there is no report on CK-loaded advanced liposomal formulations. A summary of CK-loaded nanoformulations and their advantages is shown in Table 2. In the future, the development of CK-loaded advanced liposomes may be a topic of interest in topical administration of ginsenosides. In addition, understanding the molecular pathways [132,133] and anti-tumor mechanism of ginsenosides for tumor therapy are important.

**Figure 9:**
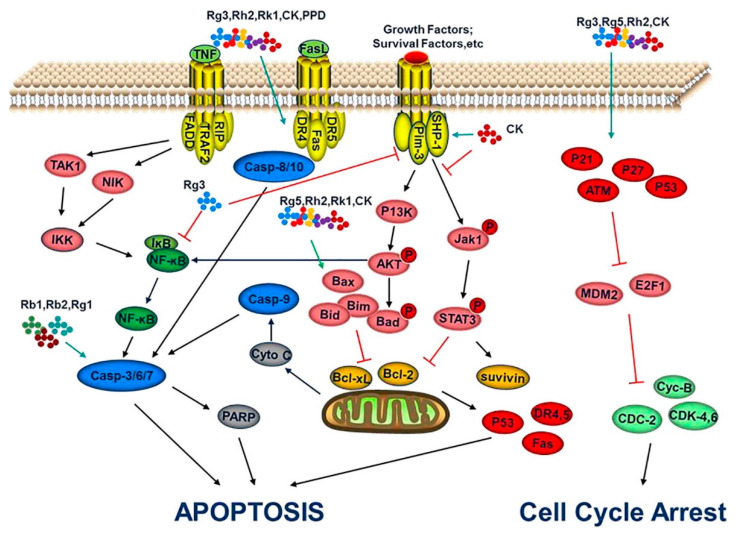
Molecular mechanism involved in tumor growth regression using ginsenosides. Reprinted with permission from Ref. [136]. 2018, Elsevier Ltd.

Ginsenosides have shown good preventive and therapeutic effects in cancer and play a complementary role in cancer therapy. Ginsenosides and their metabolites are effectively regulating various signaling pathways involved in tumor growth and metastasis [134]. Ginsenosides induce anti-cancer activity by upregulating tumor apoptosis, inhibiting the growth of cancer stem cells, regulating the tumor microenvironment and downregulating tumor angiogenesis proteins [135]. For instance, ginsenosides CK, Rg3 and Rh2 effectively upregulate the gene expression of ATM, p15, p27 and p53 and downregulate the gene expression of MDM2 which leads to the cell cycle arrest (Figure 9). In addition to the cell cycle arrest, ginsenosides induce endogenous apoptosis via tumor cell membrane proteins. Ginsenosides such as CK, Rg3, Rh2 and Rk1 upregulate the expression of Bcl-2 proapoptotic family proteins (Bad, Bid, Bim, Bax and Bak) and downregulate the Bcl-2 and Bcl-xL proteins. This leads to the mitochondrial membrane potential decrease and the release of cytochrome C, which results in the activation of caspase-9. Finally, this pathway can regulate the downstream effector molecules caspase-3/6/7 and decompose poly ADP-ribose polymerase 1 (PARP), which results in apoptosis of cancer cells.

## 4. Conclusions and Future Perspectives

This review summarized the transformation of major ginsenosides into minor ginsenosides. Special attention was paid to the preparation of ginsenoside CK, and its potential as an anti-cancer agent by loading into various nanoformulations was discussed. Ginsenosides as natural remedies have long been recognized because of their pharmacological effects. To date, the biotransformation of ginsenosides using β-glucosidase enzymes expressed in *E. coli* is the most predominant technique used to prepare minor ginsenosides because of its mild reaction conditions, selectivity and environmental-friendliness. However, *E. coli* is an inedible bacterium that limits its application of pharmaceutical and nutraceutical product development. Therefore, preparation of the β-glucosidase enzyme using a generally regarded as safe (GRAS) or edible substance is a much-needed approach in minor ginsenoside synthesis. In addition, the cost, time consumption and laborious process of ginseng production are major shortcomings in ginsenoside synthesis. To surmount these issues, synthesis of high efficiency ginsenosides using yeast cell factories could be promising and this method could be used for the mass production of minor ginsenosides.

Despite their potential, the biomedical application of ginsenosides has been severely limited because of their poor bioavailability. To improve bioavailability, studies have focused on the development of efficient nanodelivery systems and examined their different administration route to improve their bioavailability. In general, an ideal delivery system effectively maintains drugs at certain concentrations and specifically delivers drugs to the target sites (e.g., tumor), while minimizing the drug concentration in other major organs (e.g., liver, spleen, heart and kidney). Ginsenosides have long been used to treat cancer. The development of ginsenoside-loaded nano drug delivery has provided some improvement in tumor regression. In this review, representative nanoformulations such as nanoemulsion, polymeric nanoparticles and liposomes used to load CK were covered and their improvement in solubility, tumor-targetability and bioavailability were discussed. Although ginsenoside-loaded nanoformulations showed a certain prospect in tumor treatment, the anti-tumor mechanism of ginsenoside-based formulations was not clearly understood and needed additional research. Preclinical evaluation of ginsenoside-loaded nanoformulations in animal models can be a reasonable research strategy to fully understand the stability and bioavailability of ginsenosides. Furthermore, functionalization or loading of minor ginsenosides using biocompatible polymers and nanoformulations have shown promising improvement in the solubilization and tumor-targeting of ginsenosides. However, there are some issues, such as the usage of organic solvents during ginsenoside loading or chemical modifications that lead to loss of bioactivity of ginsenosides during chemical modification, which may limit their applications. Therefore, loading of ginsenosides by simple mixing into formulations (e.g., nanogels and hydrogels) may be eco-friendly and economic, and conjugation/post-functionalization of the nanocarriers with tumor-targeting functional moieties may change the perspective of ginsenoside-based nanoformulation development.

Despite some progress in the development of ginsenoside-based delivery systems, few reports showed the site-specific delivery of ginsenoside to tumor tissues. In addition, due to the lack of understanding of the anti-tumor mechanism of ginsenosides, most of the studies used a single drug and, therefore, their extent of anti-tumor activity was limited. Therefore, advanced ginsenoside-based delivery systems with tumor-targeting ligands, nanoformulations with stimuli-responsive characteristics, and combination with multiple ginsenosides, could be advanced delivery formulations that could maximize the treatment potential of ginsenosides not only in tumor treatment but also in other intractable diseases, such as stroke, Alzheimer and arthritis.

## Figures and Tables

**Table 1:** Enzymatic and microbial transformation methods for the production of CK.

Enzymes and Microorganisms	Transformation Pathways	Conditions	Time/Yield	Remarks
β-glucosidase from *M. esteraromaticum* BL21 (DE3)	Rb1 → Rd → CK	pH 7.0 and 40 °C	60 min/77%	Complete conversion of Rb1 to CK
β-glucosidase from *Caldicellulosiruptor bescii*	Rc → Rd → F2 → CK	pH 5.5 and 80 °C	180 min	The productivity of CK from Rc was 400 (µM/h)
β-glucosidase from *Lactobacillus brevis*	GypXVII → CK	pH 6.0 and 30 °C	6 h/89%	-
β-glucosidase from *Pyrococcus furiosus*	Rb1, Rb2 or Rc → Rd → CK	pH 5.5 and 95 °C	6 h/79.5%	The CK was hydrolyzed into aglycone PPD after 5 h
*Bifidobacterium* sp. Int57 from fecal microflora	Rb1 → Rd → F2 → CK	pH 5.0 and 37 °C	-	Transformation of Rb1 → CK was proceeded at higher rate.
*Arthrinium* sp. GE 17–18 from endophytic fungus	Rc → Rd → F2 → CK	pH 7.0 and 30 °C	48 h	-
*Leuconostoc citreum* LH1 from Kimchi	Rb1 → XVII → Rd → F2 → CK	pH 6.0 and 30 °C	72 h	Required long time for CK production.

**Table 2:** CK-loaded nanoformulations for cancer therapy.

Nanoformulations	Polymers	Targeting Ligand	In Vitro	In Vivo	Key Findings and Major Effect	Ref.
**Nanoemulsion**	CMC-β-CD NGs	-	A549 and PC-9 cells	A549 xenograft mouse model	CK-loaded NGs inhibited the tumor and the tumor inhibition rate was 73.8%.	[109]
**Polymeric nanoparticles**	PEG-PLGA nanoparticles	MDM2	Caco-2, LNCap, DU 145 and PC3 cells	PC3 xenograft model	GS25-loaded NPs showed good tumor growth reduction and the inhibition rate of PC3 tumor growth was 87%.	[115]
	GC–CK nanoparticles	-	HT29, HT22 and HepG2 cells	-	GC-based nanoparticles improved the solubility of CK over 100-fold and selectively delivered it into the tumor acidic environment.	[116]
	A54-PEG-DA−OCMC polymers	A54 peptide	HepG2 and Huh-7 cells	-	APD-CK nanoparticles selectively targeted liver cancer cells and effectively inhibited the proliferation of HepG2 and Huh-7 cells when compared with free CK.	[117]
**Liposomes**	TPGS and phospholipid	-	A549 cells	A549 xenograft mouse model	The CK-loaded liposomal formulation showed good anti-tumor efficacy compared with free CK.	[124]
	TPGS and PEG-PCL	-	A549 and PC-9 cells	A549 tumors	The CK-loaded formulation effectively inhibited tumor growth and the tumor inhibition rate was 52.04% ± 4.62% compared with free CK.	[125]
	DSPE-PEG2000	tLyp-1 (sequence CGNKRTR)	A549 cells	A549 tumors	tLyp-1-bearing liposomes loaded with parthenolide and CK increased tumor targeting and the anti-tumor effect.	[126]

## Data Availability

Not applicable.
